# A new objective titration procedure using Remotely Contactless Intelligent Sleep Monitoring System for the treatment of mandibular advancement device in OSAHS patient

**DOI:** 10.3389/fneur.2025.1631296

**Published:** 2025-07-18

**Authors:** Huijia Lei, Sixiang Zhu, Jing Yang, Youqing Lai, Zijing Wang

**Affiliations:** Department of Otorhinolaryngology, Beijing Jishuitan Hospital, Capital Medical University, Beijing, China

**Keywords:** Obstructive Sleep Apnea-Hypopnea Syndrome (OSAHS), Remotely Intelligent Sleep Monitoring System, mandibular advancement device, compliance, titration cycle

## Abstract

**Background:**

The key to treating Obstructive Sleep Apnea-Hypopnea Syndrome (OSAHS) by mandibular advancement device (MAD) lies in determining the optimal mandibular advancement, but current subjective titration methods are time-consuming and have poor compliance. Therefore, this study proposes an objective titration scheme based on a Remotely Contactless Intelligent Sleep Monitoring System (RCISMS) to optimize the MAD titration process, improve treatment efficiency, and enhance patient comfort.

**Methods:**

This study enrolled 60 OSAHS patients, randomly divided into a RCISMS-guided titration group (*n* = 30) and a subjective titration group (*n* = 30). Patients in the RCISMS-guided titration group used RCISMS at home to monitor AHI, which was transmitted in real-time to clinicians online for remote guidance on MAD adjustments. The subjective titration group required adjustments based on patient self-reports during clinic visits. The primary endpoint was the reduction in AHI, and secondary endpoints included titration time efficiency and improvements in subjective symptoms (Epworth Sleepiness Scale, Snoring Scale).

**Results:**

Both RCISMS-guided and subjective titration significantly reduced AHI (by 73.7 and 69.0%, respectively), with no significant difference in final AHI levels between the two groups (*p* = 0.0828). RCISMS-guided titration significantly shortened the titration cycle (27.00 ± 2.12 days vs. 45.07 ± 8.25 days, *p* < 0.0001), saving 40.01% of the time compared to subjective titration. There were no significant differences between the two groups in ESS reduction (RCISMS group 3.0 ± 1.2 vs. subjective titration group 3.3 ± 1.5, *p* = 0.3943) and Snoring VAS scores reduction (RCISMS group 3.8 ± 0.5 vs. subjective titration group 3.9 ± 0.5, *p* = 0.3306).

**Conclusion:**

The RCISMS-guided MAD titration scheme can achieve the same therapeutic effect as traditional subjective titration methods in a shorter time, while reducing the number of patient visits, improving treatment convenience and compliance, and demonstrating significant potential for clinical application.

## 1 Introduction

Obstructive Sleep Apnea-Hypopnea Syndrome (OSAHS) is a respiratory disorder characterized by the repeated occurrence of complete (lasting ≥10 s) and/or partial hypoventilation events during sleep (1, 2). Typical clinical manifestations of the condition include nocturnal snoring, excessive daytime sleepiness, and cognitive dysfunction. OSAHS has emerged as a global health concern affecting both adults and children (3, 4). Accumulating evidence indicates that OSAHS is a multifactorial disease associated with various risk factors including hypertension, vitamin D levels (5), obesity (6), insulin resistance (7), and age. Recent studies have identified congenital craniofacial anomalies as contributing factors in pediatric OSAHS (8, 9). Furthermore, sleep disturbances following the COVID-19 pandemic may exacerbate OSAHS pathogenesis (10, 11). Long-term chronic course of OSAHS can lead to irreversible damage to multiple organ systems, including the heart, brain, and kidneys (12, 13), and significantly increases the risk of severe complications such as cardiovascular diseases and metabolic syndrome (14, 15).

In clinical treatment strategies, Continuous Positive Airway Pressure (CPAP) is considered the gold standard non-surgical therapy for moderate to severe OSAHS patients (16). However, for patient’s intolerance to CPAP, the Mandibular Advancement Device (MAD) has become an important alternative treatment. MAD is commonly employed in the correction of Class II malocclusion to augment airway dimensions (17). It encompasses fixed functional appliances such as the Herbst appliance (18) and the MALU appliance (19). MAD works by progressively advancing the lower jaw to enlarge the airway space, effectively improving airway obstruction (20, 21). Compared to CPAP, MAD offers significant advantages in treatment adherence and patient tolerance, and it has become an essential approach in OSAHS management (22, 23).

The core of MAD treatment lies in determining the optimal mandibular advancement, a process achieved through titration. This involves gradually advancing the mandible as the patient adapts, until the maximum therapeutic effect is achieved or the patient cannot tolerate further adjustments (24). The traditional “subjective titration” approach relies on patient-reported symptom feedback for progressive adjustment, but it has multiple limitations: frequent clinic visits reduce adherence, and the lack of objective assessment indicators can lead to excessive titration, ultimately affecting long-term treatment efficacy (25, 26). As a result, clinical exploration has focused on titration techniques based on objective indicators, primarily including two modes: (1) Drug-Induced Sleep Endoscopy (DISE)-assisted titration, during which experienced otolaryngologists use RCMP devices under real-time endoscopic monitoring to dynamically assess airway morphology changes; (2) Polysomnography (PSG)-guided titration, combining overnight PSG monitoring with remote RCMP adjustment for precise intervention of respiratory events (16, 27). However, these methods present notable limitations: firstly, the rapid titration process lacks an adaptation phase, resulting in jaw discomfort; secondly, both approaches necessitate specialized equipment and require the involvement of trained professionals for their implementation.

Recent advancements include the development of the Sunrise system by Jean-Louis Pépin’s team, which uses a tri-axial gyroscopic chin sensor for at-home AHI monitoring. This system combines a 6-month remote doctor-patient interaction to complete MAD titration (28, 29). However, the chin sensor used in this system may interfere with patient comfort and sleep architecture, limiting its clinical application potential.

Building on this foundation, this study innovatively proposes a non-contact objective titration approach. The research team has previously developed a Remotely Contactless Intelligent Sleep Monitoring System (RCISMS), which integrates an under-mattress sleep monitor (SC-500™, Boshi Linkage Technology, Beijing, China) with an artificial intelligence analysis platform. This system can capture vital signs such as heart rate and respiration in real-time and generate detailed sleep reports that include AHI and sleep pattern. Clinicians remotely guide MAD adjustments via a cloud platform based on AHI dynamics and patient feedback until optimal efficacy thresholds or tolerance limits are achieved. This study aims to systematically evaluate the clinical application value and feasibility of RCISMS-guided objective titration in MAD treatment for OSAHS patients.

## 2 Materials and methods

### 2.1 Subjects

A total of 60 volunteers who underwent polysomnography (PSG) monitoring in the Department of Otorhinolaryngology, Beijing Jishuitan Hospital, Capital Medical University, from March to June 2024 were consecutively enrolled. General information of the participants was recorded, including age, gender, body mass index (BMI), Epworth Sleepiness Score (ESS) and Snoring VAS. This study was approved by the Medical Ethics Committee of Beijing Jishuitan Hospital, Capital Medical University (Approval No. K2023-364-00), and all participants signed informed consent forms. The severity of OSAHS is traditionally assessed using the Apnea-Hypopnea Index (AHI), which defines AHI as the number of apneas and hypopneas per hour of sleep. Based on AHI, OSAHS severity is classified as mild (5 ≤ AHI < 15/h), moderate (15 ≤ AHI < 30/h), and severe (AHI ≥ 30/h). To ensure accurate diagnosis, all patients underwent baseline PSG before the initiation of any treatment. Ear, nose, and throat (ENT) examinations, dental screenings, and assessments for inclusion and exclusion criteria were conducted. All patients were randomized to undergo one of two titration procedures: the subjective titration procedure or the new objective titration procedure using RCISMS.

Inclusion Criteria: OSAHS patients with AHI ≥ 15; Age between 18 and 70 years; Ability to provide informed consent; Refusal or non-adherence to CPAP or unwillingness to undergo upper airway surgery; No abnormalities in periodontal and temporomandibular joints (TMJ).

Exclusion Criteria: Active periodontal issues, including tooth mobility; Active temporomandibular joint dysfunction; Inadequate dental occlusion or dentition to support MAD; Patients with severe, unstable systemic diseases or mental health disorders; Enlarged tonsils (Friedman grade IV tonsils).

### 2.2 Sample size calculation

The sample size was calculated based on the primary outcome measure of AHI reduction. We anticipated an AHI reduction of approximately 70% with MAD therapy. To detect a clinically meaningful difference of 15% in AHI reduction between the RCISMS-guided and subjective titration groups, with a standard deviation of 8%, a power of 90%, and a two-sided alpha level of 0.05, a minimum of 25 participants per group was required. Accounting for a potential dropout rate of 20% during the 60-day study period, we aimed to enroll 30 participants per group, for a total of 60 participants. The sample size calculation was performed using the following formula:

*n* = 2(Z*α*/2 + Zβ)^2^ × *σ*^2^/Δ^2^. Where: *n* is the required sample size per group, Zα/2 = 1.96 (for *α* = 0.05, two-sided), Zβ = 1.28 (for 90% power), σ = 8 (estimated standard deviation of AHI reduction percentage), *Δ* = 15 (minimum clinically important difference in AHI reduction percentage). This calculation yielded 24.37 participants per group, which was rounded up to 25 and then adjusted for potential dropouts to arrive at the final sample size of 30 per group.

Ultimately, this study enrolled a total of 60 patients. Thirty patients were randomly assigned to the RCISMS-guided titration program, and 30 patients were assigned to the subjective titration program.

### 2.3 Construction of RCISMS

The system consists of an under-mattress sleep monitor SC-500™ (Identifier No. 20182071457, Boshi Linkage Technology, Beijing, China), a signal transmission network, a cloud platform, an information storage and processing workstation, an artificial intelligence analysis system, a visualization system, and an external pulse oximeter. The sleep monitor SC-500™ with a built-in electret condenser microphone (EM246ASSTM; Hakujitsu Technology Co., Tokyo, Japan) can detect and collect 0.01–10 kHz frequency domain signals. The signals can be separated accurately of vital sign information such as heart rate (0.8–1.5 Hz) and respiration (0.2–0.8 Hz), ballistocardiogram (BCG, 0.6-20 Hz) and snoring (100–500 Hz). The collected raw signals are processed using our patented comb filtering technology [Patent No. CN201310157508.8], enabling accurate separation of cardiac, respiratory, and body movement signals. Through respiratory waveform analysis and feature annotation, we identify sleep-related respiratory events while developing an intelligent computational model for the AHI. By continuously comparing with gold-standard PSG measurements and employing iterative deep learning optimization, we progressively enhance the accuracy of the AHI algorithm. The sleep pattern is based on the algorithm of BCG and consists of the total sleep time (TST), deep sleep time (DST), light sleep time (LST), rapid eye movement sleep time (REMST), the sleep efficiency and AHI (30, 31). Previous studies have proven the accuracy of this sleep monitor, using PSG as the gold standard (32, 33), and we have validated the consistency between RCISMS and PSG monitoring for AHI. When PSG diagnosed patients with moderate OSAHS, RCISMS demonstrated good agreement with PSG (Cohen’s kappa = 0.67, *p* = 0.04). For severe OSAHS cases diagnosed by PSG, RCISMS showed excellent agreement (Cohen’s kappa = 0.842, *p* = 0.001). But for mild OSAHS cases diagnosed by PSG, the consistency between RCISMS and PSG was weaker (Cohen’s kappa = 0.340, *p* = 0.046). Additionally, a signal transmission network, cloud platform, information storage and processing workstation, and visualization system are further developed, allowing sleep data from OSAHS patients undergoing MAD treatment to be remotely collected, uploaded in real-time, intelligently analyzed, and automatically generating sleep reports. The results are simultaneously presented to both the doctor and the patients.

### 2.4 Mandibular advancement device

Qualified dental specialists will conduct comprehensive assessments to determine patient suitability for MAD therapy. Key anatomical features, including mandibular protrusive capacity and temporomandibular joint function, will be evaluated. The patient will begin with habitual occlusion and then be asked to protrude the lower jaw maximally, known as the maximum protrusion (MP). Afterward, the patient will gradually retract the lower jaw and then slowly protrude again. This step is repeated three times until the position of discomfort is reached, known as the maximal comfortable protrusion (MCP). All patients in this study will receive customized, titratable MAD (Identifier No. 20232140393, Zhuhai Hanzhimei Health Technology Co., Ltd., China) treatment. The MAD used in this study is a two-piece, custom-made adjustable acrylic device that includes a calibrated micro-adjustment mechanism, with each graduation representing a 0.25 mm advancement increment. Each adjustment moves the lower jaw forward by 0.25 mm. The MAD fitting will be defined as the study starting point (0 mm mandibular advancement, baseline, Day 1), when baseline characteristics are collected. Patients will be asked to complete a Snoring visual analog scale (VAS) and ESS.

### 2.5 Subjective titration procedure

This procedure is based on the evolution of the patient’s self-reported symptoms and physical limits. Patients will be recalled to the hospital every 1–2 weeks. Depending on the patient’s responses to the family questionnaire regarding subjective changes including snoring, daytime sleepiness, tooth pain, temporomandibular joint pain, the clinician will adjust the MAD by increasing the degree of mandibular advancement by 0.5–1 mm each time, until reaching MCP, or until the patient can no longer tolerate any further mandibular advancement. The titration period will last up to 60 days.

### 2.6 RCISMS-guided titration procedure

This procedure is based on AHI data provided by the RCISMS. Patients will utilize the SC-500™ device for nightly sleep monitoring at home. This contactless under-mattress sensor continuously collects sleep-related parameters, with automated daily reports quantifying AHI values transmitted in real-time to clinicians via a secure cloud interface. Clinicians will conduct remote consultations every 3–7 days to guide MAD adjustments (0.25–1.0 mm increments) based on AHI trends and patient feedback (symptom evolution and physical limits).

**Figure 1:**
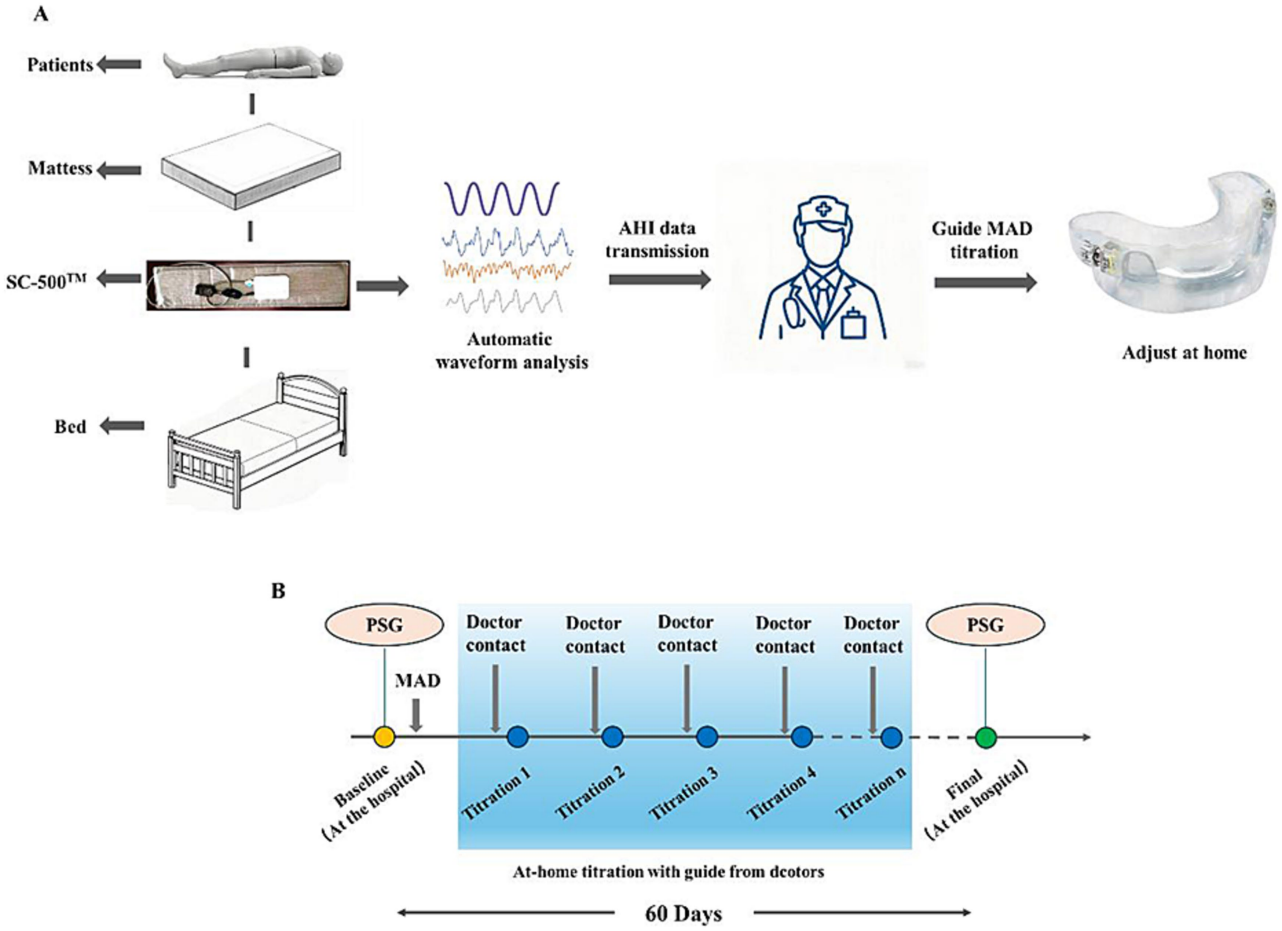
RCISMS-guided titration procedure. **(A)** The process of RCISMS-Guided titration. **(B)** The period of RCISMS-Guided titration.

In general, the degree of mandibular advancement will gradually increase until reaching MCP, or until the patient can no longer tolerate further progression. Each patient will receive individual guidance and training to make actual adjustments to the MAD at the beginning of the study. The titration period will last up to 60 days (Figure 1).

### 2.7 Outcome measures

At the completion of MAD adjustment, all patients were recalled to the hospital for PSG monitoring.

Primary Outcome: reduction in AHI based on PSG from baseline.

Secondary Outcomes: titration time efficiency and improvement in subjective symptoms. Titration time efficiency refers to the percentage of time saved within the specified period, with the specified period being 60 days. Improvement in subjective symptoms will be assessed using the ESS and snoring visual analog scale (VAS).

The ESS will be used to assess the degree of daytime sleepiness. The ESS is a self-administered questionnaire that evaluates subjective daytime sleepiness in everyday situations. The score ranges from 0 to 24, with higher scores typically indicating sleep apnea patients, suggesting a tendency for sleepiness. Details of ESS are shown in Supplementary Table S1.

The severity of snoring will be assessed using the VAS. For patients who have bed partners able to report snoring intensity, the bed partner will use a standard 10-point VAS to assess snoring. This VAS ranges from 0 to 10, where 0 means no snoring, and 10 means the bed partner leaves the room or sleeps separately. Severe snoring will be defined as a VAS snoring index of at least 7.

### 2.8 Statistical analysis

Continuous variables will be presented as mean ± standard deviation. Paired two-tailed t-tests will assess within-group changes using SPSS 26.0 (IBM Corp.). Statistical significance threshold: *p* < 0.05.

## 3 Results

### 3.1 Demographic and anthropometric data

The detailed information was presented in Table 1. The subjective titration group exhibited a mean age of 44.5 (73.3% male), with an average BMI of 27.9 and pre-titration apnea-hypopnea index (AHI) of 30.4 events/h. The RCISMS-guided titration group exhibited a mean age of 45.6 (76.7% male), with an average BMI of 28.8 and pre-titration AHI of 31.4.

**Table 1:** Baseline AHI measured by RCISMS and baseline AHI measured by PSG.

Items	Subjective titration	RCISMS-guided titration	*p*-value
Age	44.5 ± 11.3	45.6 ± 9.4	0.7307
Gender			
Male	22	23	–
Female	8	7	–
BMI	27.9 ± 5.0	28.8 ± 2.4	0.1993
AHI	30.4 ± 8.2	31.4 ± 8.8	0.6574

### 3.2 RCISMS-guided titration and subjective titration have no significant difference in reducing AHI

The subjective titration group demonstrated a baseline AHI of 30.4 ± 8.2, which decreased to 9.5 ± 2.9 post-titration, yielding a reduction of 69.0 ± 1.6%. In contrast, the RCISMS-guided titration group exhibited a baseline AHI of 31.4 ± 8.8, which was substantially reduced to 8.1 ± 3.3 post-intervention, achieving a clinically significant decrease of 73.7 ± 10.1%. There was no significant difference in the AHI before (*p* = 0.6574) and after (*p* = 0.0828) titration and in both groups. Both protocols achieved comparable post-titration AHI levels. Furthermore, the RCISMS-guided titration showed a higher percentage reduction (*p* = 0.0154) in AHI (73.7 ± 10.1%) compared to subjective titration (69.0 ± 1.6%), underscoring the therapeutic potential of RCISMS-guided titration as a viable alternative for OSAHS management (Table 2).

**Table 2:** AHI changes in subjective titration and RCISMS-guided titration.

Items	Subjective titration	RCISMS-guided titration	*p*-value
AHI before titration (events/h)	30.4 ± 8.2	31.4 ± 8.8	0.6574
AHI after titration (events/h)	9.5 ± 2.9	8.1 ± 3.3	0.0828
AHI reduction (%)	69.0 ± 1.6	73.7 ± 10.1	0.0154

### 3.3 RCISMS-guided titration significantly reduces the time required for titration

During the titration period, an average of 4.4 adjustments were required in the RCISMS-guided group compared to 4.7 in the subjective titration group. As shown in Table 3, for the first MAD adjustment, the subjective titration group took 9.20 ± 2.33 days, while the RCISMS-guided titration group took 6.40 ± 1.07 days (*p* < 0.0001). For the second MAD adjustment, the subjective titration group took 12.30 ± 1.82 days, while the RCISMS-guided titration group wore the MAD for 6.90 ± 0.99 days (*p* < 0.0001). For the third MAD adjustment, the subjective titration group took 9.30 ± 1.93 days, while the RCISMS-guided titration group took 4.83 ± 0.46 days (*p* < 0.0001). For the fourth MAD adjustment, the subjective titration group took 7.73 ± 2.38 days, while the RCISMS-guided titration group took 4.67 ± 0.71 days (*p* < 0.0001). For the fifth MAD adjustment, the subjective titration group took 6.53 ± 1.98 days, while the RCISMS-guided titration group took 4.20 ± 1.88 days (*p* < 0.0001). The RCISMS-guided titration group was able to adapt and proceed to the next adjustment in a shorter time after each MAD adjustment. Additionally, the total titration time for the RCISMS-guided titration group was significantly shorter than that of the subjective titration group. Over a 60-day period, the subjective titration program saved only 24.89 ± 13.76% of the time, while the RCISMS-guided titration program saved 55.00 ± 3.53% of the time (*p* < 0.0001).

**Table 3:** The adaptation time required for each adjustment of MAD during titration.

Number of adjustments	Subjective titration	RCISMS-guided titration	*p*-value
1	9.20 ± 2.33	6.40 ± 1.07	<0.0001
2	12.30 ± 1.82	6.90 ± 0.99	<0.0001
3	9.30 ± 1.93	4.83 ± 0.46	<0.0001
4	7.73 ± 2.38	4.67 ± 0.71	<0.0001
5	6.53 ± 1.98	4.20 ± 1.88	<0.0001
Total titration days	45.07 ± 8.25	27.00 ± 2.12	<0.0001
Time saved (%)	24.89 ± 13.76	55.00 ± 3.53	<0.0001

### 3.4 RCISMS-guided titration and subjective titration have no significant difference in the improving ESS scores and snoring VAS

The ESS was used to assess the level of daytime sleepiness, and the snoring VAS was used to evaluate the severity of snoring. As shown in Table 4, both titration protocols demonstrated statistically significant reductions in ESS scores post-intervention (RCISMS-guided titration: *p* < 0.0001; Subjective titration: *p* < 0.0001). Significant reductions were similarly observed in the snoring VAS following both titration procedures (RCISMS-guided titration: *p* < 0.0001; Subjective titration: *p* < 0.0001). But there was no significant difference between the subjective titration program and the RCISMS-guided titration program in terms of reducing ESS (*p* = 0.3943) and snoring VAS (*p* = 0.0.3306).

**Table 4:** ESS scores and snoring VAS changes in subjective titration and RCISMS-guided titration.

Items	Subjective titration	RCISMS-guided titration	*p*-value
ESS			
Before	9.8 ± 3.0	9.7 ± 3.6	>0.9999
After	6.8 ± 1.9	6.4 ± 2.3	0.7325
*p*-value	<0.0001	<0.0001	
Reduction	3.0 ± 1.2	3.3 ± 1.5	0.3943
Snoring VAS			
Before	7.6 ± 1.8	7.6 ± 1.5	>0.9999
After	3.8 ± 1.9	3.7 ± 1.8	0.7822
*p*-value	<0.0001	<0.0001	
Reduction	3.8 ± 0.5	3.9 ± 0.5	0.3306

## 4 Discussion

According to the latest guidelines (34), CPAP remains the first-line treatment for OSAHS patients, particularly for those with moderate-to-severe disease severity as determined by respiratory event frequency during sleep. However, concerns persist regarding CPAP adherence. As alternative therapeutic options, Class II occlusal correction devices including MADs demonstrate significant appeal for primary snorers and CPAP-intolerant patients (35, 36). Compared with maxillomandibular advancement surgery, these orthodontic devices offer distinct advantages of minimally invasive nature and higher patient acceptability (37). MAD has been confirmed as an effective treatment for OSAHS (38, 39), and the key factor in MAD therapy is determining the optimal mandibular advancement, which needs to be achieved through the MAD titration process (40). Currently, MAD titration often uses the traditional “subjective titration protocol” (41), which requires several months, multiple hospital visits, and MAD adjustments based on patient feedback. This process is not only time-consuming but also relies on the patient’s subjective judgment, which can easily lead to over-adjustment or under-adjustment, thus affecting treatment outcomes (42). In contrast, the RCISMS-guided titration utilizes a contactless under-mattress sleep monitor (SC-500™), which continuously monitors the patient’s AHI at home. The real-time data is uploaded to the clinician’s system, allowing for remote guidance to accurately adjust the MAD advancement, thereby avoiding over-adjustment and patient discomfort.

In recent years, several objective titration procedures have been developed, such as RCMP-based DISE-assisted titration, RCMP-based PSG-guided titration, and titration based on an automatic mandibular jaw movement (MJM) monitoring and analysis system. RCMP-based DISE-assisted titration and PSG-guided titration complete the MAD titration in a single visit (43), which saves a significant amount of time, but patients do not gradually adapt, inevitably causing noticeable mandibular discomfort. The titration method based on the automatic MJM monitoring and analysis system allows remote monitoring of the patient’s AHI and provides titration guidance, with the titration process completed at home (28). However, patients must wear a tri-axial gyroscopic chin sensor during sleep, which may affect their sleep comfort.

Currently, the internationally recognized “gold standard” for diagnosing and assessing the efficacy of OSAHS is PSG. Preliminary studies demonstrated that SC-500™ achieved comparable accuracy to PSG in monitoring the AHI, suggesting its reliability as a guidance system for MAD titration (30, 31). Furthermore, like the MJM monitoring and analysis system, RCISMS can remotely monitor the patient’s AHI data and transmit it to clinicians (44). However, RCISMS only requires placing the sleep monitor under the mattress to complete the monitoring of physiological signals, offering the advantages of convenience, comfort, and unobtrusiveness. Based on this, we propose a RCISMS-guided titration procedure. The sleep monitor is sent to the patient, RCISMS monitors and analyzes the AHI, which is synchronized in real time to the clinician. The clinician contacts the patient by phone every 3–7 days to remotely guide them in adjusting the MAD based on the AHI and patient feedback, until AHI significantly improves, symptoms resolve, or the patient is unable to tolerate further adjustments. Compared to the traditional subjective titration procedure, our results showed that the RCISMS-guided titration achieves the same level of AHI improvement without requiring patients to visit the clinic repeatedly, making MAD titration more home-based and improving patient convenience.

We also compared the time spent on both titration procedures. Patients in the subjective titration group typically choose to visit the clinic for further MAD adjustments after adapting for 7–14 days, whereas RCISMS-guided titration patients have their AHI monitored in real-time by clinicians and typically undergo the next MAD adjustment within 7 days. Whether for a single adjustment or the total titration period, RCISMS-guided titration significantly saves treatment time compared to subjective titration. The ESS and Snoring VAS before and after titration were also recorded. Both RCISMS-guided titration and subjective titration methods effectively improved ESS and Snoring VAS, further demonstrating that RCISMS-guided titration achieves the same therapeutic effect as subjective titration in about half the time.

To ensure optimal treatment adherence, a balance must be found between effective titration time, patient tolerance, and patient comfort. Compared to subjective titration and the three reported objective titration methods, RCISMS-guided titration not only does not require the wear of sensors and can be completed at home, but also allows for real-time adjustments based on feedback, ensuring that patients can gradually adapt to the treatment within a comfortable and tolerable range, greatly improving treatment tolerance and adherence, and shortening treatment time. This method can better meet the treatment needs of patients, especially for those with limited time or those who are unable to visit the clinic frequently for other reasons, making it highly appealing.

However, the current study still has limitations. Blinding design was not implemented in the trial due to the characteristics of the intervention (RCISMS requires patients to actively operate the device, and subjective titration necessitates frequent follow-up visits). Although the AHI was measured via an automated Monitoring System to reduce human bias, patient-reported outcomes (ESS/VAS) could still be affected by participants’ awareness of group assignment. Future research could optimize design through the following approaches: (1) designating an endpoint adjudication committee for blinded analysis of all data; (2) implementing a remote system with a masked interface (neither patients nor physicians have access to the actual grouping), displaying only neutral instructions. And the current study is unable to evaluate the long-term stability of MAD therapy and its potential side effects (such as chronic stress on the temporomandibular joint, temporomandibular joint disorder). Therefore, we intend to conduct a 1- to 2-year follow-up study to systematically monitor AHI, periodontal health, and quality of life, with particular emphasis on assessing relapse rates.

In summary, RCISMS-guided MAD titration procedure is an innovative, non-invasive, comfortable, and efficient treatment option that can significantly improve the treatment experience for OSAHS patients and may become one of the mainstream methods for MAD treatment in the future.

## 5 Conclusion

This study pioneers a novel RCISMS-guided MAD titration protocol, representing a paradigm shift in Obstructive Sleep Apnea-Hypopnea Syndrome (OSAHS) management. The innovative system demonstrates clinical superiority over conventional titration approaches through its integrated capacity for continuous respiratory event monitoring and artificial intelligence-enhanced real-time AHI analysis. This technological synergy enables precise dose–response calibration of MAD positioning, effectively circumventing subtherapeutic under-titration while preventing discomfort from excessive mandibular protrusion.

The automated biofeedback mechanism of RCISMS ensures therapeutic efficacy optimization through dynamic treatment parameter adjustment, concurrently addressing two critical clinical challenges: (1) mitigating treatment-abandonment risks associated with device-related discomfort, and (2) maintaining sustained therapeutic adherence through patient-centric comfort preservation. Particularly advantageous for CPAP-intolerant populations, this titration system establishes a new standard for non-invasive OSAHS intervention, offering a scientifically validated pathway for personalized precision medicine in sleep-disordered breathing management.

## Data Availability

The original contributions presented in the study are included in the article/Supplementary material, further inquiries can be directed to the corresponding author.
