# Supplementary material for: A new objective titration procedure using Remotely Contactless Intelligent Sleep Monitoring System for the treatment of mandibular advancement device in OSAHS patient
Source: Front Neurol. 2025 Jul 18;16:1631296. doi: 10.3389/fneur.2025.1631296 (PMC12314197; doi:10.3389/fneur.2025.1631296)
Supplement: Supplementary file 1 [file Table_1.docx]

Table S1. **Epworth Sleepiness Scale (ESS)**

| Situation | Score |
| --- | --- |
| Sitting and reading |  |
| Watching TV |  |
| Sitting in a car (when stopped) |  |
| Sitting and doing nothing (e.g., in a waiting room) |  |
| Traveling by car as a passenger for a long trip |  |
| Traveling by plane (e.g., in a two-hour flight) |  |
| Sitting and talking to someone |  |
| Resting quietly, e.g., reading a book or being alone |  |
| Total |  |

For all situations:

**Never doze** = 0 points

**Slight chance of dozing** = 1 point

**Moderate chance of dozing** = 2 points

**High chance of dozing** = 3 points
